# Supplementary material for: Characterization of the artisanal fishing communities in Nepal and potential implications for the conservation and management of Ganges River Dolphin (Platanista gangetica gangetica)
Source: PeerJ. 2016 Jan 12;4:e1563. doi: 10.7717/peerj.1563 (PMC4715443; doi:10.7717/peerj.1563)
Supplement: Supplemental Information 2 [file peerj-04-1563-s002.docx]

| **Gear Type** | **Gear Name** | **General Description** | **Mesh-Size** | **Scope of Use** |
| --- | --- | --- | --- | --- |
| Gillnet | Maha Jaal | A long simple net with small weights distributed around its bottom edge. | Mesh size ranges from 0.5 to 1 mm | Mainly in ponds or narrow river channel |
|  | Bagaune Jaal | A net that is dragged or hauled across a river or along the bottom of a lake or sea. The fishing depth of this net can be adjusted by adding weights to the bottom. | Varies in size based on target species. | Narrow channel river |
|  | Tiyari Jaal | A net that is used by two people in small wooden boat where one hand is hitting the water with paddle and other is catching fish. | Varies in size based on target species. | Narrow river channel with slow water current |
|  | Maha Jaal | A long simple net with small weights distributed around its bottom edge which is hold by two persons on two ends. | Mesh size ranges from 0.5 mm to 1 mm | Mainly in ponds or narrow river channel  by two persons or more |
|  | Paat or current Jaal | Drift netting (locally called *Current or Paat jaal),* is a fishing technique where drift nets hang vertically in the water column and drift with the current without being anchored to the bottom. The nets are kept vertical in the water by floats attached to a rope along the top of the net and weights attached to another rope along the bottom of the net. | Varies in size based on target species. | Main river channel |
|  |  |  |  |  |
|  |  |  |  |  |
| **Cast Net** | Phekuwa jaal or Haate jaal | A cast net (locally known as *Phekuwa jaal*), also called a throw net. It is a circular net with small weights distributed around its edge. | Ranges from 1.2 to 3.6 m (4- 12 ft) | Ponds, lake, or river |
| **Other** | Ghumauwa or  Khaap Jaal | A kind of lift net that has an opening that faces upwards and submerged to a desired depth, and then lifted or hauled from the water manually or mechanically. | Ghumauwa or  Khaap Jaal | Stream or river |
|  | Dadhiya | Locally knitted bamboo stick is placed in flowing water path as the obstruction for the fishes. | Dadhiya | Stream or river |
|  | Pakhure Jall | A hand net, also called a scoop net or dip net, (locally *pakhure jaal*) is a net or mesh basket held open by a hoop. A hand net with a long handle is often called a *dip net*. The basket is made of wire or nylon mesh. The hand net is sometimes used to help land a fish it is called a *landing net*. | Mesh size varies from 1 to 2 mm; it can be larger depending on the target species. | Shallow water |
